# Development of an Asthma Exacerbation Risk Prediction Model for Conversational Use by Adults in England

**DOI:** 10.2147/POR.S424098

**Published:** 2023-10-04

**Authors:** Constantinos Kallis, Rafael A Calvo, Bjorn Schuller, Jennifer K Quint

**Affiliations:** 1 National Heart and Lung Institute, and School of Public Health, Imperial College London, London, UK; 2 Dyson School of Engineering, Faculty of Engineering, Imperial College London, London, UK; 3 Department of Computing, Faculty of Engineering, Imperial College London, London, UK

**Keywords:** asthma, exacerbation, risk prediction, electronic healthcare records

## Abstract

**Background:**

Improving accurate risk assessment of asthma exacerbations, and reduction via relevant behaviour change among people with asthma could save lives and reduce health care costs. We developed a simple personalised risk prediction model for asthma exacerbations using factors collected in routine healthcare data for use in a risk modelling feature for automated conversational systems.

**Methods:**

We used pseudonymised primary care electronic healthcare records from the Clinical Practice Research Datalink (CPRD) Aurum database in England. We combined variables for prediction of asthma exacerbations using logistic regression including age, gender, ethnicity, Index of Multiple Deprivation, geographical region and clinical variables related to asthma events.

**Results:**

We included 1,203,741 patients divided into three cohorts to implement temporal validation: 898,763 (74.7%) in the training sample, 226,754 (18.8%) in the testing sample and 78,224 (6.5%) in the validation sample. The Area under the ROC curve (AUC) for the full model was 0.72 and for the restricted model was 0.71. Using a cut-off point of 0.1, approximately 27 asthma reviews by clinicians per 100 patients would be prevented compared with a strategy that all patients are regarded as high risk. Compared with patients without an exacerbation, patients who exacerbated were older, more likely to be female, prescribed more SABA and ICS in the preceding 12 months, have history of GORD, COPD, anxiety, depression, live in very deprived areas and have more severe disease.

**Conclusion:**

Using information available from routinely collected electronic healthcare record data, we developed a model that has moderate ability to separate patients who had an asthma exacerbation within 3 months from their index date from patients who did not. When comparing this model with a simplified model with variables that can easily be self-reported through a WhatsApp chatbot, we have shown that the predictive performance of the model is not substantially different.

## Introduction

Asthma is a chronic inflammatory respiratory disease, common across Europe, with ~30 million diagnosed cases among children and adults aged <45 years. In the United Kingdom, over 5.4 million people have asthma,^1^ accounting for over 65,000 hospital admissions and 1000 deaths annually.^2^ Asthma is defined as a heterogeneous disease, usually characterized by chronic airway inflammation. It is defined by the history of respiratory symptoms such as wheeze, shortness of breath, chest tightness and cough that vary over time and in intensity, together with variable expiratory airflow limitation.^3^

Asthma exacerbations range from milder events, which interrupt daily life and work productivity, to more severe and life-threatening exacerbations. In 2016/17, >75,000 people spanning all age groups experienced an asthma exacerbation, which warranted hospitalisation. People who have exacerbations are at risk of having further exacerbations.^4^ Asthma exacerbations were defined as worsening of symptoms,^5^ which required a short course of oral corticosteroids (OCS), an A&E visit, or a hospitalisation. However, whether there are specific factors that affect the likelihood of having an exacerbation of asthma and how this may differ from one individual to the next has not been fully investigated.^6–9^ Improving accurate risk assessment of exacerbations, and reduction via relevant behaviour change among people with asthma could save lives and dramatically reduce health care costs. In order to be maximally effective, behaviour change interventions need to incorporate risk assessment in ways that people will use and chatbots (eg, on WhatsApp) are a promising approach.^10^

There are a number of papers in scientific literature that describe risk models to predict asthma exacerbations.^11–15^ As it was necessary to implement a relatively simple model that would be incorporated into the chatbot described previously, it was necessary to develop a new prediction model using the minimum number of risk factors possible whilst being as methodologically robust as possible. These risk factors should also be suitable for data collection on WhatsApp and readily known by patients. Readily available models not designed with these specifications are unlikely to be suitable for our project. Therefore, our objective was to develop a personalised risk prediction model for asthma exacerbations by exploring factors collected in routine healthcare data which could be used to inform development of a chatbot service. Our aim was to develop the simplest model possible, without losing accuracy, and then to integrate this into a software platform that provides the risk modelling feature to any conversational system. Conversational systems, such as a WhatsApp chatbot aim to address the early-care gap by offering a new type of low-cost, and scalable personalised risk assessment that is followed-up with recommendations for action.^16^

## Methods

Pseudonymised primary care electronic records from the Clinical Practice Research Datalink (CPRD) Aurum database built in February 2022 were obtained. CPRD Aurum contains information on individuals registered at general practices in England and includes information on consultations, clinical diagnoses, therapies prescribed, and referrals to secondary care.^17^ Linked data from Hospital Episode Statistics (HES) and mortality data from the Office of National Statistics (ONS) were provided for this study by CPRD/NHS (National Health Service) Digital for patients in England.

### Study Population

We included a cohort of people with asthma aged 18 years and older based on the first date of having a primary care contact related to asthma during their follow-up period (index date). A patient’s follow-up period is the time period during which predictors and outcome variables can be defined using available data. Start of follow-up was defined as the latest date of the following: being 18 years old, study start date (January 1st 2010), being eligible for linkage with HES and being registered at CPRD practice for at least a year. End of follow-up period was defined as the earliest date of the following: death, CPRD registration end date, CPRD practice last collection date, end of study date (December 31st 2019), being 100 years old.

### Predictor Variable Definitions

We combined variables for prediction of asthma exacerbations using logistic regression. We used demographic variables including age, gender, ethnicity, Office for National Statistics (ONS) Index of Multiple Deprivation (IMD) quintile and geographical region in England.

We also added clinical variables directly related to asthma events and treatment including the number of asthma exacerbations in the previous 12 months from index date, the number of SABA (Short-Acting Beta Agonist) and the number of ICS (Inhaled Corticosteroids) canister prescriptions during the last 12 months. We also included a categorical predictor corresponding to GINA steps (1–5) criteria that are used to select initial controller treatment in adults and adolescents with a diagnosis of asthma. We combined these categories related to asthma control level based on Global Initiative for Asthma (GINA) steps based on GINA 2020 guidelines^18^ with a category that corresponded to patients with SABA only prescriptions (following^5^). Finally, we added a category for patients with no medications related to asthma during the last 12 months and GP review for asthma in the last 12 months. Furthermore, we added information on relevant co-morbidities such as prior atopy (either eczema, food allergy or allergic rhinitis), prior history of Gastro-oesophageal reflux disease (GORD), Chronic Obstructive Pulmonary Disease (COPD), anxiety and depression. We also included information on past and current behaviour related to smoking and vaping and vaccination for influenza in the 12 months prior to index date.

We have added vaccination for two reasons. First, this is an important management metric that is well recorded in primary care. Second, this information gave us an idea as to how people engage with and are or are not proactive about their healthcare. Vaccination positive history is not a risk factor but a demonstration of appropriate care that is more likely given at GINA step 5 and inappropriately not given at earlier steps.

### Outcome Definition

We defined our outcome variable for each patient as a binary indicator for having at least one asthma exacerbation recorded within 90 days from their study start. Asthma exacerbation events could have been recorded in either primary care data records, HES Admitted Patient Care (APC) or HES Accident and Emergency (A&E) data. We defined a short course of OCS as a prescription for oral prednisolone not prescribed on the same day as an annual asthma review. More specifically, disease codes for asthma exacerbations recorded in primary care were used in combination with secondary care codes corresponding to either hospitalisation, A&E admission or use of systemic steroids. Whilst the gold standard definition of an asthma exacerbation includes worsening of symptoms,^5^ this is not something that we could routinely capture in the data and so we used a definition that has been used in these data multiple times previously.^19–23^

### Statistical Analysis

#### Logistic Regression

Initially, we created a logistic regression model using all candidate predictors described previously. A logistic risk model was built as this type of model could provide a risk equation that would be easy to implement and transparent for risk model users that require an explanation for their risk prediction. However, given the data would be collected by a chatbot that can talk to people over WhatsApp (or other messaging platforms), we created an additional logistic regression by restricting candidate predictors to variables that can be easily self-reported by application users. Subsequently, we compared the two models using a variety of statistical performance criteria to check if the simpler model with restricted list of predictors would be comparable with the full model with all candidate predictors.

### Area Under the Curve

The initial statistical criterion for training our logistic regression model was the Area under the ROC curve (AUC) as it is an established measure of risk model discrimination between high and low risk patients. According to,^24^ an AUC that is at least 0.7 is considered as acceptable so we used this threshold as the minimum AUC criterion for our training sample. To estimate the Area under the ROC curve (AUC), we implemented Stata command “somersd” with an appropriate transformation to obtain Harrell’s c-statistic that estimates AUC.

### Net Reclassification Improvement

To include predictors in our model that would result in improved accuracy, we used Net Reclassification Improvement (NRI) as it is closely linked to AUC.^25^ NRI is the estimated percentage of improvement by comparing the percentage of patients with probabilities that changed in the correct direction and the corresponding percentage of patients with probabilities who changed in the wrong direction. The corresponding z-statistic was obtained by dividing the estimated NRI with the corresponding standard error. We estimated the NRI in the training sample to evaluate candidate predictors in terms of determining who had a high probability of asthma exacerbation within 90 days for each predictor separately. Each predictor with significant NRI contribution was then included in a multivariate logistic regression in the next step of our analysis.

### Calibration

An additional statistical criterion we implemented was model calibration (comparing actual and predicted outcome proportions after grouping the data). We utilised Hosmer-Lemeshow statistic as it is a well-known statistical measure of model goodness of fit and calibration. For estimates of this statistic in large samples, we also considered that very small deviations from well-calibrated predicted values can lead to statistically significant results in terms of rejecting good calibration. Calibration was evaluated using the Stata command “hl” that allowed implementation of the corresponding Hosmer-Lemeshow test using the maximum number of groups possible, subject to the constraint that all expected values exceed 5 and adjustment of degrees of freedom (d.f.) for training sample. In addition, we implemented Stata command “pmcalplot” to assess calibration and to estimate Calibration-in-The-Large (CILT) and Calibration Slope.

As grouping for Hosmer-Lemeshow statistic is arbitrary and p-values depend on the extent of lack of calibration and sample size,^26^ we also estimated additional calibration metrics such as Calibration-In-The-Large (CILT) and calibration slope for additional checking. Using calibration metrics, we updated the predicted probabilities in training and validation to improve calibration in these samples.^27^

Model updating to improve calibration for patients in testing sample was achieved using the predicted linear predictor (LP) from each of the two training sample logistic regression models described earlier. Using each model LP, we implemented an additional logistic regression model that included LP as the only predictor using testing dataset patients to estimate the intercept and slope that were utilised at the next step to obtain the updated linear predictor (ULP).

Subsequently, we multiplied LP with the estimated slope and added the estimated intercept (intercept + slope x LP) to obtain the updated linear predictor (ULP):

$${\mathrm{ULP=intercept + slope\,x\,LP}}$$

Finally, we applied the anti-logit transformation to ULP to obtain the updated predicted probabilities (UPP):

$${\mathrm{UPP=(exponential}}\left({{\mathrm{ULP}}}\right){\mathrm{/}}\left({{\mathrm{1+exponential}}\left({{\mathrm{ULP}}}\right)}\right)$$

### Decision Curve Analysis

Measures such as AUC and calibration cannot provide information on the clinical utility of our prediction model.^28^ For this purpose, we utilised Decision Curve Analysis (DCA) to estimate Net Benefit (NB). Following the implementation of a suitable cut-off point for predicted probabilities to define high risk patients, NB was estimated using a combination of true positive and false positive patients. This type of analysis required us to define an appropriate cut-off point based on a combination of clinical expertise and the estimate of the prevalence of the outcome variable.

We implemented temporal validation as an approximation to prospective external validation. To be specific, we used data from earlier years (index dates between 1/1/2010 – 31/12/2015) to build a prediction model (training sample) and later years (index dates between 1/1/2016 and 31/12/2018) as temporally proximal (testing) sample. The final year (1/1/2019 – 31/12/2019) was used as temporally distal (validation) sample.

We have chosen to use temporal validation as an approximation to prospective design. In this way, we can check whether a model built on current data would be robust enough for future applications in subsequent years.

All analyses were performed using STATA statistical software, version 17.

### Ethics Approval

CPRD has NHS Health Research Authority (HRA) Research Ethics Committee (REC) approval to allow the collection and release of anonymised primary care data for observational research [NHS HRA REC reference number: 05/MRE04/87]. Each year CPRD obtains Section 251 regulatory support through the HRA Confidentiality Advisory Group (CAG), to enable patient identifiers, without accompanying clinical data, to flow from CPRD contributing GP practices in England to NHS Digital, for the purposes of data linkage [CAG reference number: 21/CAG/0008]. The protocol for this research was approved by CPRD’s Research Data Governance Group via submission of an eRAP for MHRA Database Research (protocol number 22_001728) and the approved protocol was made available to the journal and reviewers during peer review. This study is based in part on data from the Clinical Practice Research Datalink obtained under license from the UK Medicines and Healthcare products Regulatory Agency. The data are provided by patients and collected by the NHS as part of their care and support. The interpretation and conclusions contained in this study are those of the author/s alone. Linked pseudonymised data were provided for this study by CPRD. Data are linked by NHS Digital, the statutory trusted third party for linking data, using identifiable data held only by NHS Digital. Select general practices consent to this process at a practice level with individual patients having the right to opt-out.

This study complies with the Declaration of Helsinki.

## Results

The total number of research acceptable patients was 40,933,535 and the percentage of UK population coverage was 13,354,913 out of 67,081,000 (19.91%).^29^ The total number of patients eligible for linkage was 37,503,753 and the total number of GP practices was 1489. The percentage coverage of UK general practices was 1358 out of 8178 (16.61%).

We included 1,281,102 patients who met our inclusion criteria. We excluded a small number of patients sequentially with missing information on smoking status (n=3357), geographic region (n=14,110) Index of Multiple Deprivation (n=1,409), ethnicity (n=43,371) and the predictor combining GINA steps, or no medications during the last 12 months (n=15,114). The remaining 1,203,741 patients were divided into three cohorts to implement temporal validation: 898,763 (74.7%) in the training sample, 226,754 (18.8%) in the testing sample and 78,224 (6.5%) in the validation sample.

### Descriptive Analysis

The first step in our analysis was to investigate in the training sample predictor differences between those with the outcome (at least one asthma exacerbation in 90 days from index date) and those without this outcome. 93,625 patients (10.4%) had at least one asthma exacerbation in 90 days from index date. Descriptive analysis and related test statistics from Chi-square tests for categorical variables and from Mann–Whitney non-parametric tests for continuous variables are shown in Tables 1 and S1.

**Table 1:** Descriptive Statistics and Comparisons by Outcome Status in Training Sample (n=898,763)

Outcome	Without Outcome (n=805,138)		With Outcome (n=93,625)		Statistic value	Test p-value
At least one asthma exacerbation in 90 days from index date	n	%	n	%		
**Predictors**						
Age (median, IQR)	46	33–62	53	39–68	−83.4	<0.001
Female	467,576	58.1	61,036	65.2	1800.0	<0.001
Number of SABA canisters in last 12 months (median, IQR)	1	0–4	3	1–7	−104.6	<0.001
Number of ICS canisters in last 12 months (median, IQR)	1	0–4	3	0–7	−100.2	<0.001
Prior history of atopy (eczema, food allergy, allergic rhinitis)	292,001	36.3	34,344	36.7	6.3	0.012
Smoking status					1800.0	<0.001
Never smoked	228,308	28.4	20,720	22.1		
Ex-smoker	362,203	45.0	43,950	46.9		
Current smoker	214,627	26.7	28,955	30.9		
Vaping status					184.8	<0.001
Never vaped	790,716	98.2	91,356	97.6		
Ex-vaper	383	0.1	67	0.1		
Current vaper	14,039	1.7	2202	2.4		
Asthma exacerbations in last 12 months (median, IQR)	0	0–0	1	0–2	−250.0	<0.001
Prior history of Gastro-oesophageal reflux disease (GORD)	69,326	8.6	11,097	11.9	1100.0	<0.001
Prior history of Chronic Obstructive Pulmonary Disease (COPD)	62,687	7.8	17,506	18.7	12,000.0	<0.001
Prior history of anxiety	111,580	13.9	16,343	17.5	889.1	<0.001
Prior history of depression	130,359	16.2	19,881	21.2	1500.0	<0.001
At least one GP review for asthma in last 12 months	144,357	17.9	24,034	25.7	3300.0	<0.001
GINA steps/SABA only/No medications combination					51,000.0	<0.001
No medications	187,828	23.3	14,243	15.2		
SABA only	413,364	51.3	34,810	37.2		
GINA step 1	37,053	4.6	3092	4.2		
GINA step 2	4374	0.5	657	0.7		
GINA step 3	19,348	2.4	1381	1.5		
GINA step 4	96,833	12.0	14,220	15.2		
GINA step 5	46,338	5.8	24,412	26.1		
Influenza vaccination in last 12 months	368,755	45.8	52,868	56.5	3800.0	<0.001

Compared with patients without an exacerbation, patients who exacerbated were older, more likely to be female and were prescribed more SABA and ICS canisters in the preceding 12 months. Patients with and without an exacerbation had comparable rates of atopy. People who exacerbated within 90 days were more likely to be smokers or vapers and had more asthma exacerbations in the last 12 months. People who exacerbated were also more likely to have prior history of GORD, COPD, anxiety or depression, more likely to have had a GP review for their asthma in the last 12 months and live in very deprived areas. Finally, people who exacerbated were far more likely to have more severe disease (be at GINA step 5) and more likely to have been vaccinated for flu in the last 12 months from index date. Put together, our findings of asthma patients at higher risk of exacerbations being older, female, with greater GINA severity and prior exacerbations have all been identified previously as associated co-morbidities.

### NRI

The results from NRI analysis are presented in decreasing NRI value (percentage improvement) order in Table 2.

**Table 2:** NRI Analysis Results

Outcome	Univariate NRI		
At Least One Asthma Exacerbation in 90 Days from Index Date	NRI (%)	z-Statistic	p-value
**Predictors**			
Asthma exacerbations in last 12 months	64.7	187.3	<0.001
GINA steps/SABA only/No medications combination	45.4	131.5	<0.001
Number of SABA canisters in last 12 months	32.0	92.7	<0.001
Number of ICS canisters in last 12 months	31.9	92.3	<0.001
Age	24.4	70.7	<0.001
Prior history of Chronic Obstructive Pulmonary Disease (COPD)	21.8	63.2	<0.001
Influenza vaccination in last 12 months	21.3	61.8	<0.001
At least one GP review for asthma in last 12 months	15.5	44.8	<0.001
Gender	14.2	41.2	<0.001
Prior history of depression	10.1	29.2	<0.001
Geographical region in England	9.9	28.7	<0.001
Smoking status	8.5	24.7	<0.001
Index of Multiple Deprivation Quintile	7.5	21.8	<0.001
Prior history of anxiety	7.2	20.8	<0.001
Prior history of Gastro-oesophageal reflux disease (GORD)	6.5	18.8	<0.001
Ethnicity	3.0	8.7	<0.001
Vaping status	1.3	3.7	<0.001
Prior history of atopy (eczema, food allergy, allergic rhinitis)	0.8	2.4	0.016

All candidate predictors were significant at 5% level based on NRI, therefore, all predictors considered were included simultaneously in multivariate logistic regression. The number of asthma exacerbations in the last 12 months had the highest NRI amongst candidate predictors.

### Logistic Regression

Using candidate predictors in Table 2, we created two logistic regression models using patients in the training sample. The first was a model with all predictors (full model; Table 3) and the second a model with predictors that were chosen based on feasibility of self-reporting within a WhatsApp application (restricted model; Table 4).

**Table 3:** Full Logistic Regression Model

Outcome	Odds Ratio	95% CI	p-value
At Least One Asthma Exacerbation in 90 Days from Index Date			
**Predictors**			
Asthma exacerbations in last 12 months	1.65	1.64–1.66	<0.001
Female	1.27	1.25–1.29	<0.001
Number of SABA canisters in last 12 months	1.03	1.03–1.03	<0.001
Number of ICS canisters in last 12 months	0.99	0.99–1.00	<0.001
Smoking status			
Never smoked (Reference category)	Ref.	Ref.	Ref.
Ex-smoker	1.04	1.02–1.06	<0.001
Current smoker	1.21	1.18–1.23	<0.001
Age	1.00	1.00–1.00	<0.001
Vaping status			
Never vaped (Reference category)	Ref.	Ref.	Ref.
Ex-vaper	1.25	0.94–1.64	0.119
Current vaper	1.10	1.05–1.16	<0.001
Prior history of atopy (eczema, food allergy, allergic rhinitis)	1.02	1.01–1.04	0.002
Prior history of Gastro-oesophageal reflux disease (GORD)	1.05	1.02–1.07	<0.001
Prior history of Chronic Obstructive Pulmonary Disease (COPD)	1.25	1.22–1.28	<0.001
Prior history of anxiety	1.06	1.04–1.08	<0.001
Prior history of depression	1.14	1.11–1.16	<0.001
At least one GP review for asthma in last 12 months	1.33	1.31–1.35	<0.001
London (reference category)	Ref.	Ref.	Ref.
North East	1.54	1.47–1.60	<0.001
North West	1.27	1.24–1.31	<0.001
Yorkshire	1.39	1.33–1.45	<0.001
East Midlands	1.37	1.30–1.45	<0.001
West Midlands	1.27	1.24–1.31	<0.001
East of England	1.22	1.17–1.28	<0.001
South East	1.23	1.20–1.27	<0.001
South West	1.33	1.29–1.37	<0.001
Index of Multiple Deprivation Quintile			
1 (least deprived) (reference category)	Ref.	Ref.	Ref.
2	1.02	1.00–1.05	0.096
3	1.04	1.02–1.07	0.001
4	1.04	1.01–1.06	0.006
5 (most deprived)	1.06	1.04–1.09	<0.001
Ethnicity			
White (reference category)	Ref.	Ref.	Ref.
Black	1.01	0.97–1.06	0.537
South Asian	1.22	1.18–1.25	<0.001
Mixed	1.03	0.96–1.10	0.457
Other	1.04	0.96–1.12	0.334
GINA steps/SABA only/No medications combination			
No medications (reference category)	Ref.	Ref.	Ref.
SABA only	0.80	0.79–0.82	<0.001
GINA step 1	0.95	0.91–0.98	0.006
GINA step 2	1.18	1.08–1.29	<0.001
GINA step 3	0.68	0.69–0.72	<0.001
GINA step 4	1.12	1.09–1.16	<0.001
GINA step 5	1.52	1.47–1.57	<0.001
Influenza vaccination in last 12 months	0.92	0.90–0.94	<0.001

**Table 4:** Partial Logistic Regression Model

Outcome	Odds Ratio	95% CI	p-value
At least one asthma exacerbation in 90 days from index date			
**Predictors**			
Asthma exacerbations in last 12 months	1.73	1.72–1.74	<0.001
Prior history of Chronic Obstructive Pulmonary Disease (COPD)	1.37	1.34–1.41	<0.001
Number of SABA canisters in last 12 months	1.02	1.02–1.03	<0.001
At least one GP review for asthma in last 12 months	1.31	1.29–1.33	<0.001
Female	1.26	1.24–1.28	<0.001
Prior history of depression	1.14	1.12–1.16	<0.001
Number of ICS canisters in last 12 months	1.01	1.01–1.01	<0.001
Smoking status			
Never smoked (Reference category)	Ref.	Ref.	Ref.
Ex-smoker	1.04	1.02–1.06	<0.001
Current smoker	1.21	1.18–1.23	<0.001
Prior history of anxiety	1.06	1.04–1.08	<0.001
Age	1.00	1.00–1.00	<0.001
Prior history of Gastro-oesophageal reflux disease (GORD)	1.05	1.03–1.08	<0.001
Vaping status			
Never vaped (Reference category)	Ref.	Ref.	Ref.
Ex-vaper	1.27	0.97–1.68	0.086
Current vaper	1.12	1.07–1.18	<0.001
Prior history of atopy (eczema, food allergy, allergic rhinitis)	1.02	1.01–1.04	0.004

Based on these results, we can conclude that in addition to NRI results, there is further evidence that candidate predictors are related to the outcome variable in our regression models after adjusting for other predictors.

In Tables 5 and S2, descriptive statistics are shown for the outcome variable and candidate predictors for each sample type (training, testing and validation).

**Table 5:** Descriptive Statistics and Comparisons by Sample Type

Variable	Training Sample		Testing Sample		Validation Sample		Statistic Value	Test p-value
	N*	%	N	%	n	%		
**Outcome**								
At least one asthma exacerbation in 90 days from index date	93,625	10.4	25,537	11.3	8545	10.9	145.0	<0.001
**Predictors**								
Age (median, IQR)	47	33–63	42	28–58	43	29–60	10,277.9	<0.001
Female	528,612	58.8	131,503	58.0	45,073	57.6	82.4	<0.001
Number of SABA canisters in last 12 months (median, IQR)	1	0–4	1	0–3	1	0–3	5887.6	<0.001
Number of ICS canisters in last 12 months (median, IQR)	1	0–4	0	0–3	1	0–3	11,109.5	<0.001
Prior history of atopy (eczema, food allergy, allergic rhinitis)	326,345	36.3	66,553	29.4	23,203	29.7	4800.0	<0.001
Smoking status							20,000.0	<0.001
Never smoked	249,028	27.7	92,290	40.7	31,592	40.4		
Ex-smoker	406,153	45.2	75,476	33.3	26,191	33.5		
Current smoker	243,582	27.1	58,988	26.0	20,441	26.1		
Vaping status							734.5	<0.001
Never vaped	882,072	98.1	220,782	97.4	76,081	97.3		
Ex-vaper	450	0.1	158	0.1	47	0.1		
Current vaper	16,241	1.8	5814	2.6	2096	2.7		
Asthma exacerbations in last 12 months (median, IQR)	0	0–0	0	0–0	0	0–1	294.8	<0.001
Prior history of Gastro-oesophageal reflux disease (GORD)	80,423	9.0	15,789	7.0	6019	7.7	986.8	<0.001
Prior history of Chronic Obstructive Pulmonary Disease (COPD)	80,193	8.9	14,580	6.4	6455	8.3	1500.0	<0.001
Prior history of anxiety	127,923	14.2	34,739	15.3	13,693	17.5	716.1	<0.001
Prior history of depression	150,240	16.7	33,243	14.7	12,524	16.0	566.0	<0.001
At least one GP review for asthma in last 12 months	168,391	18.7	31,804	14.0	10,124	12.9	4000.0	<0.001
GINA steps/SABA only/No medications combination							15,000.0	<0.001
No medications	202,071	22.5	62,429	27.5	21,145	27.0		
SABA only	448,174	50.0	110,236	48.6	37,027	47.3		
GINA step 1	40,955	4.6	17,014	7.5	7112	9.0		
GINA step 2	5031	0.6	2371	1.1	1026	1.3		
GINA step 3	20,729	2.3	3217	1.4	887	1.1		
GINA step 4	111,053	12.4	21,095	9.3	6849	8.8		
GINA step 5	70,750	7.9	10,392	4.6	4178	5.3		
Influenza vaccination in last 12 months	421,623	46.9	82,969	36.6	29,574	38.0	9200.0	<0.001

**Notes**: *For categorical variables, numbers (n) and percentages (%) are shown and in addition Chi-square statistic values with the corresponding p-values for the comparison between sample types are given. For continuous variables, medians and Interquartile Ranges (IQR) are shown and Kruskal–Wallis non-parametric statistic values and p-values are given.

Compared with testing and validation sample patients, training sample patients were less likely to have an asthma exacerbation within 90 days from their asthma index date, were slightly older and almost equally likely to be female. The number of SABA and ICS canisters prescribed during the last 12 months before index date were comparable (Table 5). People in the training sample compared with the other two samples were more likely to have prior atopy (either eczema, food allergy or allergic rhinitis), less likely to be never smokers and more likely to be ex-smokers and almost equally likely to be current smokers. In terms of vaping status, patients in the training sample were less likely to be current vapers and more likely to be never vapers. The number of previous asthma exacerbations in the last 12 months in each sample was comparable. The difference between the training sample and the other two samples in terms of outcome prevalence is purely coincidental. The magnitude of this difference is small and these samples are fairly comparable.

### AUC

For the training sample, the AUC for the full model was 0.72 (95% CI 0.72–0.72) and for the restricted model was 0.71 (95% CI 0.71, 0.71). As expected, the AUCs for both models in testing and validation samples were reduced compared with the corresponding AUCs in the training sample. In the testing sample, the full model AUC was 0.70 (95% CI 0.69–0.70) and for restricted model was 0.68 (95% CI 0.68–0.69). Finally, in the validation sample, the AUC for validation sample was 0.69 (95% 0.68–0.70) for full model and 0.67 (95% CI 0.67–0.68) for restricted model.

To provide indicative estimates for sensitivity and specificity, we used 0.1 (10%) as a suitable cut-off point. In the training dataset, for the restricted model, sensitivity was 54.1% (95% CI 53.7–54.4%) and specificity was 78.6% (95% CI 78.5–78.7%). For the full model, using the same cut-off point, sensitivity was 54.3% (95% CI 53.9–54.6%) and specificity was 78.7% (95% CI 78.6–78.8%).

### Calibration

For the training sample, Hosmer-Lemeshow statistic showed acceptable goodness of fit and acceptable calibration. HL statistic was 116.9 (99 d.f., p-value = 0.083) for the restricted model and 116.5 (100 d.f., p-value=0.098) for the full model. The corresponding CILT was 0.0 and slope was 1.0 for both restricted and full model and indicated that observed rates and expected rates were sufficiently close and these models were well calibrated.

For testing sample, calibration based on Hosmer-Lemeshow statistic was comparable with the results for training sample only for full model. HL statistic was 205.1 (100 d.f., p-value < 0.001) for restricted model and 71.8 (100 d.f., p=0.985) for full model. CILT was 0.183 and slope was 0.872 for restricted model and the corresponding statistics for full model were respectively 0.191 and 0.896.

For the restricted model, the intercept was −0.097 and the slope was 0.872 and for the full model the corresponding values were −0.038 and 0.896 respectively. For both models, the corresponding updated predicted probabilities CILT estimates were 0.0 and slope estimates were 1.0 indicating that the updated probabilities were well calibrated compared with the actual outcome rates in the testing sample.

Similar results to the testing sample were found based on analysis of validation sample. HL statistic was 163.4 (100 d.f., p < 0.001) for restricted model and 86.4 (99 d.f., p=0.814) for full model. For restricted model, CILT was 0.101 and slope was 0.802 and for full model CILT was 0.108 and slope was 0.829. Similar to the training sample, re-calibration of the models might be required if the objective of model implementation is to estimate individual patient probabilities that match on aggregate level the actual outcome rates.

Following the same procedure described earlier to update the predicted probabilities, in the validation sample the restricted model intercept was −0.322 and the corresponding slope was 0.802. For the full model, intercept and slope were −0.257 and 0.829 respectively. As with the updated probabilities in the testing sample, the updated predicted probabilities CILT estimates were 0.0 and slope estimates were 1.0 for both models.

### DCA

Based on outcome prevalence and clinical expertise, 0.1 (10%) was selected as a suitable cut-off point for model predicted probabilities. In the training sample, 93,625 out of 898,763 people with asthma (10.4%) were identified as high-risk for 3-month asthma exacerbations. Based on this cut-off point, there were 50,609 true positive and 172,397 false positive patients using predicted probabilities from restricted model. The corresponding predicted probabilities from full model using the same cut-off point had as a result 50,795 true positive and 171,243 false positive patients. Net Benefit was estimated to be equal to 0.035 (3.5%) from both models. This means that there were 3.5 net detected 3-month asthma exacerbations per 100 prediction model users. We also estimated the net reduction in interventions (ie, referral of high-risk patients to GPs or to respiratory clinicians for clinical review for asthma). Based on patients from the training sample, it was estimated that for a cut-off point of 0.1 from our restricted prediction model, 27.3 interventions per 100 patients would be prevented compared with a strategy that all patients are regarded as high risk. The corresponding full model net reduction in interventions of 27.6 means that the full model has slightly better clinical utility compared to the restricted model.

For testing sample patients, the reductions in intervention per 100 patients were 17.4 for restricted model and 17.7 for full model. Finally, for validation sample patients, the corresponding reductions in interventions were 18.7 for both restricted and full model, suggesting that both models would be expected to be clinically useful compared with alternative strategies such as sending no patients or all patients to GPs for clinical reviews to prevent possible asthma exacerbations.

## Discussion

Using information available from routinely collected electronic healthcare record data, we have developed a model that has moderate ability to separate patients who had an asthma exacerbation within 3 months from their index date from patients who did not. When comparing this model with a simplified model with variables that can easily be self-reported through a WhatsApp chatbot, we have also shown that the predictive performance of the model is not substantially different. The modifiable predictors most strongly associated with an exacerbation event in our study were treatment related (increasing number of SABA prescriptions), smoking and vaping. Better management of the co-morbidities associated (depression, anxiety, COPD, GORD and atopy) may also improve outcomes. Our finding of ICS prescriptions being associated is likely a marker of disease severity and this association did not hold in the multivariable models.

Similar to other studies, we found that an exacerbation event in the year prior was the predictor most strongly associated with a subsequent exacerbation event.^30^ A recent paper using machine learning to predict asthma exacerbations using electronic healthcare record data found that age, long-acting beta agonist prescriptions, high dose inhaled corticosteroids or oral corticosteroids were risk factors for exacerbation events but that the model was not improved by adding spirometry. Similar to our study, one study exploring exacerbation risk at routine visits^31^ found that medication use and in particular adherence was associated with subsequent exacerbation events. We were not able to capture adherence data in our study, only the prescription of medications.

Multiple studies have focussed on paediatric populations^32^ or those with more severe asthma^33^ rather than a general asthma population.^34^ Or, they have focussed on factors that are not as easily modifiable to reduce risk.^35–37^ We deliberately chose factors that could be easily captured in routine healthcare data and that could be accurately entered into a chatbot by an individual without too much burden. Studies have also suggested that longer term follow up is needed given the relatively low rate of exacerbations in people with asthma and several studies have been unable to develop a model with acceptable accuracy to be clinically useful.^38^ We were keen to focus on a model to look at relatively short term risk so that in future work we can look to mitigate that risk.

Following the Global Initiative for Asthma, GINA^18^ strategy, asthma control is best supported with assessments in two separate domains: “future risk” including exacerbations as described previously, and “symptom control”. We have included both of these separately in the development of our chatbot which is currently being tested. To date, we have shown that it is possible to choose a subset of variables, and a shorter-term outcome with several modifiable risk factors, to be incorporated into a WhatsApp chatbot.

Subsequent steps to our analysis would include external validation of our model using suitable data from other databases. We also envisage to validate the clinical utility of WhatsApp chatbot in preventing asthma exacerbations.

## Strengths and Limitations

We had a very large sample size, representative of the general asthma population making results applicable across an adult asthma population. Our results may have been different if we had included a paediatric population. However, given the large sample size, we need to take into account that even small differences can result in statistically significant results. We have not tested the model in terms of reducing subsequent exacerbation events or tried to implement it into clinical practice but will do so as part of the larger project.^16^ We are also unable to comment on the extent to which healthcare professionals entering the data have been trained or the quality of the visit, eg, the quality of the interaction at the asthma annual review. We are not able to comment on adherence, only the prescription of treatments. It is also possible that we missed exacerbation events that went untreated. Finally, our prediction models were not created to provide unbiased causal or treatment effects but to generate an overall risk estimate based on the combination of predictors in our models. Therefore, the odds ratio for variables from the models should not be used as treatment or causal effect estimate.

Eosinophils^38^,^39^ and T2 comorbidities^40^ were not included as predictors in our models as information on these predictors is not recorded consistently in primary care records. In addition, this information is not something that is readily known by all people with asthma. The purpose of our risk prediction model was not to be as complex as possible, or to replace some of the complex prediction models in existence, but rather to develop as simple a model as possible, that could be used by patients interacting with a WhatsApp chatbot. Our outcome definition does not match the standard definition for asthma related exacerbations as patients will not always meet this definition and not all of this information is available in electronic healthcare record data.^41^ However, our definition has been used in previous studies using CPRD data. Whilst we acknowledge that use of Electronic Medical Records (EMR) has limitations, we do not believe that use of these data in this way is not appropriate. Indeed, there have been many risk prediction models using CPRD data that have been developed and we have tried to be as robust as possible in our design, even using temporal validation to approximate prospective study design.

Our prediction model includes modifiable risk factors taking into account non-modifiable risk factors such as age and gender. As our model is built to identify high risk individuals, modifiable and non-modifiable risk factors were included to obtain sufficient level of accuracy. Our study was not aimed at finding new risk factors. Our aim was to create an accurate app mainly based on risk factors identified in published literature.

We have not undertaken external validation in our analysis. Once it is possible to obtain access to additional samples, it would be possible to validate our results from temporal validation that we used as a proxy to prospective external validation.

## Conclusion

We have developed a model that has moderate ability to separate patients who had an asthma exacerbation within 3 months from their index date from patients who did not and have incorporated this simplified model into a WhatsApp chatbot for further testing.

## Data Sharing Statement

Data are available on request from the CPRD. Their provision requires the purchase of a license, and this license does not permit the authors to make them publicly available to all. This work used data from the version collected in May 2021 and has clearly specified the data selected within each Methods section. To allow identical data to be obtained by others, via the purchase of a license, the code lists will be provided upon request. Licenses are available from the CPRD (http://www.cprd.com): The Clinical Practice Research Datalink Group, The Medicines and Healthcare products Regulatory Agency, 10 South Colonnade, Canary Wharf, London E14 4PU. The data accessed complied with CPRD data protection and privacy regulations, terms and conditions.
